# X-inactive specific transcript (XIST) can determine sex differences in cardiovascular drug responses: focus on RNA therapeutics

**DOI:** 10.3389/fphar.2025.1716863

**Published:** 2026-01-12

**Authors:** Timur O. Yarovinsky, Vinod S. Ramgolam, Iva R. Knezevic, Nina S. Stachenfeld, Jeffrey R. Bender

**Affiliations:** 1 Yale Cardiovascular Research Center, Section of Cardiovascular Medicine, Department of Internal Medicine, Yale University School of Medicine, New Haven, CT, United States; 2 Department of Immunobiology, Yale University School of Medicine, New Haven, CT, United States; 3 TargetSite Therapeutics, New Haven, CT, United States; 4 Department of Obstetrics, Gynecology and Reproductive Sciences, Yale University School of Medicine, New Haven, CT, United States

**Keywords:** cardiovascular diseases, miRNA, RNA therapeutics, sex as a biological variable (SABV), X chromosome inactivation (XCI), XIST (X-inactive specific transcript)

## Abstract

Genetic, hormonal, anatomical, and environmental factors underlie sex differences in the prevalence and progression of cardiovascular disease and responses to therapeutics. The presence of two X chromosomes in the female genome decreases susceptibility to X-linked recessive disorders but imposes the need for random X chromosome inactivation as an epigenetic mechanism controlling gene dosage. Long non-coding RNA XIST is essential for transcriptional repression of genes on inactive X chromosome but may also act as a miRNA sponge for post-transcriptional regulation of gene expression and a scaffold for RNA binding proteins that provoke autoimmune responses. These features draw attention to XIST as an important drug development target by design or as an off target, including novel RNA therapeutics for genetic and cardiovascular diseases. Based on the extensive literature analysis, we postulate the hypothesis that XIST can determine sex differences in cardiovascular drug responses and propose several criteria for use in developing or evaluating responses to RNA therapeutics for cardiovascular disease in women. We hope that implementation of those criteria in the process of RNA therapeutics development may be helpful in reducing the risks of adverse effects in women.

## 1 Introduction: sex as a biological variable and X chromosome inactivation in cardiovascular diseases

Cardiovascular diseases (CVD) remain the leading cause of death in men and women worldwide and require improved approaches to diagnostics, treatment, and drug development (Hong, 2022; Mensah et al., 2023; Martin et al., 2025). Sex differences in anatomy and physiology of cardiovascular system, hormonal influences, genetic and environmental risk factors result in variable prevalence, presentation, responses to therapy, and prognosis of CVD (Regensteiner et al., 2015; Mehta et al., 2016; Hibino et al., 2025; Kim et al., 2025). Women display major differences from men in drug pharmacokinetics and pharmacodynamics, in part due to variable oral drug absorbance and bioavailability, distribution, metabolism, and clearance (Madla et al., 2021). Since adverse drug effects and associated hospitalizations are more common in women (Madla et al., 2021), drug developers should consider additional measures for adjusting the clinical trial design and the protocols for reporting adverse effects to regulatory agencies.

Drug development and regulatory oversight evolved through a complex and iterative process responding to tragic failures and setbacks. The most well-known example is thalidomide that was widely prescribed and used by pregnant women for anxiety, insomnia, and morning sickness. As a result, more than 10,000 children, mostly in Europe, Canada, and Australia were born with severe birth defects and abnormalities. The FDA refusal to approve thalidomide, due to the opinion and strong character of the FDA pharmacologist Frances Oldham Kelsey, allowed the US to minimize the harm from the use of thalidomide by pregnant women. However, because of this crisis, the FDA issued a guidance in 1977 that excluded women of reproductive potential from Phase 1 and Phase 2 clinical trials, unless they had a life-threatening condition. Although the policy was reversed in 1993 by the FDA and US Congress, which started requiring inclusion of women in clinical trials, significant gaps in our knowledge of women’s health and female under-representation in the clinical trials persist up to date.

Currently, most regulatory agencies, such as the United States Federal Drug Agency (FDA) and the European Medicines Agency (EMA), as well as the funding agencies, such as the United States National Institutes of Health (NIH), require inclusion of women in clinical trials, determination of new drug toxicity, pharmacokinetics and pharmacodynamics in both sexes in preclinical and clinical studies, and consideration of sex as a biological variable in federally funded basic and translational research unless scientifically justified otherwise. However, women have been under-represented in clinical trials: women enrollment was 38.2% in the 740 cardiovascular clinical trials completed between 2010–2017 (Jin et al., 2020) and 41% in the 1,079 cardiovascular clinical trials conducted between 2017–2023 (Rivera et al., 2025). Similarly, sex omission and male bias continue to plague basic and translational cardiovascular research (Daniel Ramirez et al., 2017; Kim et al., 2021). Correcting these disparities is necessary for improving health outcomes and is also likely to produce a great return on investments and major economic benefits.

A number of guidelines for best practices of considering sex in research, drug development and repurposing may help investigators with the study design, data collection, analyses, and interpretation of the results (Miller et al., 2017; Madla et al., 2021; Fisher et al., 2022; Ryan et al., 2024; Sutherland and Carter, 2024; Usselman et al., 2024). However, insufficient characterization of women-specific pathways and targets, pregnancy, lactation, and socio-economic disparities continue to impede full implementation of sex as a biological variable in responses to new therapeutic modalities (Singh and Swarup, 2025).

Dimorphism of sex chromosomes (XX vs. XY) is a major genetic determinant of sex development and characteristics. Males typically inherit a single Y chromosome, with its ∼100 protein coding genes and ∼400 pseudogenes and complex repeat structures, and a single copy of X chromosome, with estimated 860–1,000 protein coding genes and several hundred (400–800) pseudogenes (Ross et al., 2005; Rhie et al., 2023). Y chromosome-linked *SRY* gene (location: Yp11.2) determines testis development and male sex determination (Sim et al., 2008). Individuals with *SRY* deletions develop as females despite the 46,XY karyotype. The mosaic loss of the Y chromosome in leukocytes or disruption of the Y chromosome-linked histone demethylase *UTY* is associated with increased risk of heart failure in males (Horitani et al., 2024).

Females are usually endowed with two X chromosomes but display mosaicism with respect to the X chromosome usage due to random X chromosome inactivation (XCI or lyonization after the geneticist Mary F. Lyon who discovered the phenomenon). Yet, at least 15% of the genes on X chromosome escape inactivation in females and may be expressed at higher levels than in males (Carrel and Willard, 2005). The increased gene dosage effects for pattern recognition receptor or costimulatory molecules may contribute to higher susceptibility of females to autoimmune diseases or drugs (Lu et al., 2007; Aguado et al., 2022). Moreover, the X chromosome mosaicism and XCI expand the range of the phenotypes of X-linked recessive disorders in females. On one hand, the XCI may enhance the impact of mutations expressed from the active X chromosome at a cellular level. On the other hand, mosaicism may diminish the phenotype of mutations at the whole organism level through selection of the cells expressing the healthy allele. This process may be detected as skewed XCI and illustrated by several examples of X-linked recessive disorders with cardiac involvement (Table 1). In some cases, skewed XCI is associated with pathogenic gene expression and increased prevalence of autoimmune diseases in females. One example is primary Sjögren’s syndrome, which is considered the most female-predominant example (Whitacre, 2001): allelic skewing in minor salivary gland-derived mesenchymal stromal cells resulted in altered protein localization of several disease-associated proteins such as TSPAN6 and CHST7 (Shaw et al., 2022). Thus, the chromosomal dimorphism and epigenetic regulation of X chromosome may determine the variations in male and female susceptibility to autoimmune and X-linked recessive disorders.

**TABLE 1:** Examples of X-linked recessive cardiovascular diseases with likely involvement of XCI.

Disease	Gene and location	Description	Penetrance in females and XCI involvement	References for the involvement of XCI
Fabry disease	*GLA*, Xq22.1	Systemic disease due to deficient activity of lysosomal α-galactosidase (GLA), patients with residual GLA activity develop left ventricular hypertrophy, with or without renal failure	Variable; XCI significantly impacts the phenotype	Echevarria et al. (2016)
Barth syndrome	*TAFAZZIN*, Xq28	Dilated cardiomyopathy due to loss of cardiolipin in the inner mitochondrial membrane and dysfunction of oxidative phosphorylation	Exceptionally rare, likely due to masking by severely skewed XCI	Ørstavik et al. (1998), Cosson et al. (2012)
Duchenne muscular dystrophy	*DMD*, Xp21.2-p21.1	A muscular dystrophy with cardiac involvement	Rare; no clear correlation with XCI	Sumita et al. (1998)
Becker muscular dystrophy	*DMD*, Xp21.2-p21.1	A muscular dystrophy with cardiac involvement	Variable; symptomatic carriers have skewed XCI of the healthy allele	Viggiano et al. (2017)
Cardiac valvular dysplasia	*FLNA*, Xq28	Dystrophy of the cardiac valves	Variable; XCI	Kyndt et al. (2007)

X-inactive specific transcript (XIST) is a broadly expressed spliced long non-coding RNA that mediates the stochastic inactivation of one of the two X chromosomes in somatic cells. Its gene (*XIST*) is located within the region of X chromosome called the X inactivation center and is essential for the initiation and spread of X-inactivation. Mutations in the *XIST* promoter cause familial skewed X inactivation (Plenge et al., 1997; Pugacheva et al., 2005). Besides playing the key role in X-inactivation, XIST may act as a sponge for miRNAs, thus showing impact on gene expression beyond the X-linked genes in female cells.

Inhibitors of XIST expression and activity are in active development for applications in X-linked inherited diseases and cancer. In this manuscript, we postulate that XIST represents an important pharmacological target that should be considered in drug development for cardiovascular disease since it is a major epigenetic regulator of gene expression in females, a broad antagonist of miRNAs, and a potential trigger of inflammatory and autoimmune diseases in females. In the interest of space and clarity, we focus on the burgeoning field of RNA therapeutics delivering mRNA and microRNA agonists or antagonists and their applications to CVD. Since XIST is a very large lncRNA acting as a transcriptional silencer of X-linked genes as well as a sponge for miRNA targeting genes on X and somatic chromosomes, we suggest specific measures that prospective drug developers may consider in rational design of novel RNA therapeutics, predictive assessment of off-target activities and adverse effects, as well as possibilities for drug repurposing. In this work, we ponder the questions of whether XIST can change female responses to RNA therapeutics in development and call the investigators and drug developers to discuss what approaches and evidence are essential for designing RNA therapeutics impacting or avoiding XIST biology. We hope that the key points of this paper can help improve the efficacy and safety of RNA therapeutics targeting cardiovascular disease in women as well as in Klinefelter syndrome patients (biologically male with extra X chromosome, i.e., XXY karyotype).

## 2 X chromosome inactivation and XIST determine sex differences in cardiovascular and related diseases

### 2.1 XIST as a mediator of X chromosome inactivation

X chromosome inactivation is a process of random inactivation of one of the two X chromosomes in somatic cells during early embryonic development that controls the dosage of most but not all X-linked genes. XIST RNA is essential for X chromosome dosage compensation in eutherian mammals occurring in temporally and species- and cell lineage-variable mechanisms (Sahakyan et al., 2018; Alfeghaly and Rougeulle, 2025).^1^

In mice, rats, and voles imprinted XCI initiates in preimplantation female embryos at the four to eight-cell stage and involves inactivation of paternally inherited X chromosome (Xp), while keeping active the maternally inherited X chromosome (Xm) (Sahakyan et al., 2018). Xp remains repressed in the extraembryonic tissues (placenta) but is transiently reactivated in the epiblast cells at the blastocyst stage, which give rise to the embryo proper. The second wave of XCI randomly inactivates either Xp or Xm and maintains their repressed status in all descendent cells resulting in a mosaic pattern of X-linked expression in adult mice. In mice, the antisense lncRNA Tsix acts as a negative regulator of Xist, thereby influencing which of the two X chromosomes is maintained in the active state (Sado, 2017). Tsix and another lncRNA Xite apparently mediate the process of X chromosome counting and are essential for X chromosome counting and establishing XCI (Lee, 2005). Most other placental mammals show no evidence of imprinted XCI but rely on random XCI for extraembryonic and embryonic cell lineages (Alfeghaly and Rougeulle, 2025).

Several mouse strains targeting Xist gene locus were generated by homologous recombination or CRISPR/Cas9-mediated gene targeting to create Xist-null phenotype, conditional or inducible knockouts and deletions or other mutations in different Xist exons (Csankovszki et al., 1999; Turner et al., 2002; Nesterova et al., 2003; Savarese et al., 2006; Senner et al., 2011). These experimental models helped with establishing the role and structural requirements for XIST in XCI. At least one attempt to generate CRISPR/Cas9-mediated deletion of XIST in a pig model has been reported (Savarese et al., 2006), however the model has not been characterized nor used in any follow up studies. Given the difference between rodents and other placental animals in the mechanisms of XCI in extraembryonic tissues and challenges for modeling CVD in rodents, additional genetic models targeting XIST in large mammals are needed.

**FIGURE 1:**
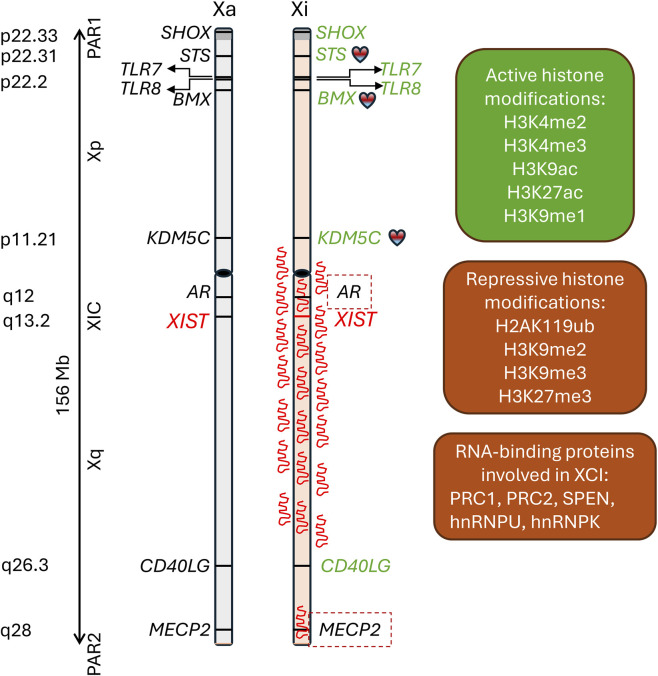
A map of X chromosome inactivation (XCI) and escape. Transcription of the X-linked lncRNA XIST from the X chromosome inactivation center (XCI) proceeds to spreading on and coating the X chromosome in cis, resulting in inactivation of one of the two X chromosomes in females and in Kleinfelter syndrome 47,XXY males. XIST recruitment of the polycomb repressive complex (PRC2 and PRC1) and interactions with other RNA-binding proteins results in accumulation of the repressive histone modifications, DNA hypermethylation, and transcriptional silencing of most genes on the inactive X chromosome (Xi) followed by compacting the Xi into Barr bodies (not shown). While XCI provides adequate dosage for most Xi-linked genes, expression of disease-causing alleles from the active X chromosome (Xa) makes females vulnerable to X-linked inherited diseases (e.g., germline mutations in MECP2 are lethal in males and cause Rett syndrome in females). A subset of genes on Xi escape XCI (examples are shown in green), resulting in excessive gene dosage (double in 46,XX females when compared to 46,XY males, or even higher in trisomy X, i.e., 47,XXX). Many genes that escape XCI are clustered in the distal regions, especially of the short arm of the X chromosome and near or at the pseudoautosomal region (PAR1). The XCI escape increases the risks for autoimmune and inflammatory diseases in females and may contribute to the metabolic manifestations of Kleinfelter syndrome. Notably, XCI escape of several genes (marked with the ailing heart icon) has been linked to sex differences in cardiovascular diseases and responses of females to statins and other drugs.

Human *XIST* gene (https://www.omim.org/entry/314670) is located on the X chromosome long arm at Xq13.2 in the X inactivation center (Figure 1) and spans slightly more than 32 kb. Its transcript is a subject to regulated alternative splicing of nine exons, producing multiple splicing variants of exceptionally long (17–19.3 kb) non-coding (i.e., untranslated) RNA (Hong et al., 2000; Stork et al., 2019). Comparative genome analyses indicate that *XIST* gene evolved in eutherian mammals likely though disruption of a protein-coding gene *LNX3* and combination of transposable elements (Duret et al., 2006; Elisaphenko et al., 2008). XIST RNA exon 1 RNA includes repeat regions A, F, B, C, and D, of which the A-repeat located at the exon’s 5′ end is highly conserved and is necessary and sufficient to induce transcriptional silencing (Wutz et al., 2002). XIST RNA exon 7 includes repeat region E and is essential for interaction with the RNA binding protein hnRNP U and XIST nuclear retention (Yamada et al., 2015).

Human preimplantation female embryos and human naïve embryonic stem cells have both X chromosomes transcriptionally active during the first week of development and rely on X chromosome dampening (XCD) for regulation of gene dosage (Sahakyan et al., 2018). In this process, transcription of XIST is initiated from both X chromosomes at the preimplantation stage and the transcriptional activity of both X chromosomes is transiently attenuated by XIST recruiting polycomb repressive complex (PRC) in cooperation with the RNA-binding protein SPEN (Alfeghaly et al., 2024). The polycomb repressive complex-associated histone modifications (H3K27me3 and H2AK119ub) mark the X chromosome regions associated with XIST in most cells of female preimplantation embryos but are absent in the highly expressed genes that escape X chromosome dampening (Alfeghaly et al., 2024).

The random XCI in human embryos and in non-human primates is initiated around the implantation period (day 7) but is not complete by day 12 (Alfeghaly and Rougeulle, 2025). The X inactivation center and X chromosome counting are clearly important in establishing the random XCI, which is dependent on XIST RNA spreading and coating of the inactive X chromosome (Maduro et al., 2016). However, TSIX is not likely to be essential for X chromosome counting since it is truncated in humans and not found in many other eutherian mammals displaying random XCI (Alfeghaly and Rougeulle, 2025). The identity and the roles of other factors that suppress XIST on the active X chromosome are still under intense investigation (Aksit et al., 2024).

Spreading, localization, and recruitment of XIST on the inactive X chromosome appears to be dependent on interaction with the RNA binding proteins SPEN, hnRNP U, and hnRNP K (Chu et al., 2015; Maduro et al., 2016; Boeren and Gribnau, 2021). Established random XCI is characterized by compacting the inactive X chromosome into Barr body, where the DNA is heavily methylated and is associated with the repressive histone marks (H2AK119ub, H3K9me2, H3K9me3 and H3K27me3). The genes throughout the affected regions of the inactive X chromosome are effectively repressed, essentially without any evidence of transcriptional activity, and the inactivation status of the X chromosome is faithfully passed on to the progeny cells indefinitely. Thus, XIST-mediated random XCI enables compensation of the gene dosage for the majority of X chromosome-linked genes with one of the two X chromosomes being inactive at a roughly equal proportions among the cells.

Skewed XCI is defined based on the evidence of more than 75%–80% cells showing preferential inactivation of one of the X chromosomes (Minks et al., 2008; Echevarria et al., 2016). Skewed XCI is often found in female carriers of mutations for X-linked recessive diseases affecting males, such as Barth syndrome. It is likely that skewed XCI is the result of selection against the cells with the active X chromosome expressing the mutated gene (Ørstavik et al., 1998). Preferential activation of the paternal X chromosome was reported in several families and associated with muscular dystrophy or X-linked Wiskott-Aldrich syndrome (Parolini et al., 1998; Sumita et al., 1998). Furthermore, recent evidence shows that aging and other selection biases occurring after XCI is established skews XCI from a completely random pattern and may contribute to increased risks of cancer or atherosclerotic cardiovascular diseases (Roberts et al., 2022).

In mice (genus *Mus*), the X chromosome element *Xce*, which is located within the chromosome X inactivation center and in proximity to the *Xist* gene, has at least five different alleles (a,b,c,d,e) that control propensity to primary XCI skewing in F1 hybrids (Calaway et al., 2013). In humans, detailed analyses of XCI patterns in epithelial and hematopoietic cells of more than 500 mother-neonate pairs did not reveal a single heritable genetic locus for skewed XCI (Bolduc et al., 2008). Yet, other studies identified a mutation within the promoter region of *XIST* gene was shown to result in non-random X-chromosome inactivation (Plenge et al., 1997), thus further supporting the notion that XIST is essential for random XCI.

Although the gene dosage control through XCI is essential for balancing female development and health in general, females carrying a disease-causing allele on the X chromosome may be partially protected, compared to males, but still suffer due to the mosaic pattern of X-linked gene expression. Rett syndrome is a rare (1:10,000 to 1:20,000 live births) neurodevelopmental disorder that is observed almost exclusively in females as arrested development at 6–18 months of age (loss of behavioral skills, learning disabilities, seizures, microcephaly). The disease is caused by loss-of-function germline mutations in *MECP2* gene that are usually embryonically lethal in 46,XY hemizygous males. The gene product, methyl-CpG binding protein two is an important transcriptional regulator of multiple genes, with *BDNF* being one of the most studied target, and is essential for neuronal morphology and synaptic function (Liyanage and Rastegar, 2014). A study with monozygotic twins with Rett syndrome suggested that skewed XCI may impact the severity of the disease (Ishii et al., 2001). As we describe further in Section 4, there is promise of achieving cure of Rett disease through restoration of the normal *MECP2* copy from the inactive X chromosome by modulation of XIST expression and/or XCI.

Spreading of nuclear XIST beyond the inactive X chromosome under various conditions, such as XIST overexpression or loss of the transcription factor YY1 acting as XIST tether to the nucleation center, suggested that XIST may bind to the autosomal chromosomes (Jeon and Lee, 2011). XIST binding to and repression of ∼100 autosomal genes has recently been reported in mouse embryonic stem cells and embryonic fibroblasts (Yao et al., 2025). The functional consequences of XIST binding to autosomal genes will have to be determined further if XIST is further explored as a therapeutic target.

### 2.2 X chromosome inactivation escape

At least 15% of X chromosome-linked genes escape from X chromosome inactivation (Carrel and Willard, 2005; Balaton and Brown, 2016). X chromosome inactivation escape is generally defined when the expression from the inactive X chromosome constitutes at least 10% of the expression level of the active X chromosome. X chromosome inactivation escape may occur constitutively or in response to developmental or environmental cues. Although the expression levels for the escaped gene on the inactive X chromosome are usually lower than from the corresponding alleles on the active X chromosome, the inactivation escape contributes to the phenotypic differences between males and females: it can protect females from X-linked genetic diseases or increase risks of autoimmune disorders. For example, the extra copies of the X-chromosome linked genes encoding single stranded RNA sensors *TLR7*, *TLR8*) or immune regulators (*CD40LG*, *CXCR3*) lead to increased sensitivity of women to lupus and other autoimmune diseases (Lu et al., 2007; Wang et al., 2016; Souyris et al., 2018; Syrett et al., 2019; von Hofsten et al., 2024).

X chromosome pseudoautosomal regions (segments at the tip of chromosome arms) and the region Xp22 on the short arm harbors many genes that escape XCI (Figure 1). The escaped genes retain active histone marks such as H3K4me2, H3K4me3, H3K9ac, H3K27ac, and H3K9me1, but lose the repressive marks, such as H3K27me3 (Balaton and Brown, 2016). In contrast to the inactive regions of the X chromosome that are coated by XIST, the escaped genes are not associated with XIST (Balaton and Brown, 2016). The difference between the inactive and escaped genes in the DNA methylation status of the CpG motifs has been used to develop highly sensitive assays for detection of clonal X-inactivation in blood cells. The easiest and most commonly used is the HUMARA assay, which takes advantage of the highly polymorphic (CAG)n repeat in the first exon of human androgen receptor gene *AR* (Allen et al., 1992). The HUMARA assay applications are limited to a single gene, thus more comprehensive assays fox XCI escape have been developed (Swierczek et al., 2012; Musalkova et al., 2015).

Several genes that escape XCI have been causatively linked to CVD and/or differential sensitivity of females to drugs. Constitutive X-inactivation escapee gene *KDM5C* encoding a histone demethylase was also recently described as a key contributor to higher prevalence in statin-induced glucose intolerance and myopathy in women (Zhang et al., 2024). Escape of *BMX* (Bmx nonreceptor tyrosine kinase) and *STS* (steroid sulfatase) genes from XIC appears to promote female myofibroblast activation and expression of α-smooth muscle actin in response to endothelin-1 and plasminogen activator inhibitor 1 (Aguado et al., 2022). These findings may partially explain the sex dimorphism of aortic valve stenosis, but also suggest that females, but males, are likely to respond to ibrutinib, a BMX inhibitor, and/or irosustat, an STS inhibitor (Aguado et al., 2022). It is tempting to speculate whether these *KDM5C*, *BMX*, and *STS* could be selectively targeted by RNA therapeutics, such as antisense oligonucleotides (ASO).

### 2.3 X chromosome inactivation and escape in X chromosome dosage variations

Excess (47,XXX in females with triple X syndrome or 47,XXY in males with Kleinfelter syndrome) or loss (45,X in females with Turner syndrome) of X chromosome have detrimental effects on human health resulting in increased morbidity and mortality. Triple X syndrome (trisomy X) is the most common chromosomal abnormality (∼1 per 1,000 females). Some individuals are asymptomatic or mildly affected, while others develop tall stature, epicanthal folds (monolid eyes), hypotonia (low muscle tone), clinodactyly (abnormal finger curvature), premature ovarian failure, seizures and cognitive disabilities (Tartaglia et al., 2010). In triple X syndrome patients, all but one of the X chromosomes are inactivated, but some of the X-linked genes are likely to escape XCI and contribute to the phenotype of trisomy X patients (Ottesen et al., 2010). In support of this hypothesis, *SHOX* gene was found to constitutively escape XCI, resulting in increased expression of the homeobox transcription factor associated with increased body height (Ottesen et al., 2010). Decreased methylation of X-linked genes *AMOT*, *HTR2C*, *IL1RAPL2*, *STAG2*, *TCEANC*, *ZNF673* in triple X syndrome patients compared to 46,XX karyotype may indicate incomplete XCI, albeit its impact on the transcript levels appears buffered (Nielsen et al., 2020). Thus, the search for other genes escaping XIC in trisomy continues.

Kleinfelter syndrome is associated with the 47,XXY karyotype, which represents the most frequent chromosomal aberration in males (∼150 per 100,000 males) (Groth et al., 2013). More than 90% of patients are infertile, have small testes, no sperm cells in the semen, and have high levels of follicle-stimulating and luteinizing hormones but low levels of testosterone. In many cases, other clinical manifestations of XXY males may be similar to XY males; hence definite diagnosis of Kleinfelter syndrome is based on karyotyping. Similar to 46,XX females, one of the two X chromosomes in Kleinfelter syndrome is randomly inactivated (Kinjo et al., 2020). XCI escape is likely to increase the gene dosage for metabolic regulators and to be responsible for the abdominal obesity and insulin resistance, albeit the specific genes responsible for development of those traits and other features of Kleinfelter syndrome have not been clearly identified.

XCI may impact penetrance or severity of X-linked recessive diseases in 46,XX females through silencing of either mutant or healthy allele. However, XCI is either absent in 45,X monosomy or its protective effects are reduced in females with X chromosome deletions impairing the healthy gene allele (Cosson et al., 2012). Another example are deletions of the pseudoautosomal region affecting *SHOX* gene that have been causatively linked with the short stature and Turner syndrome (Rao et al., 1997).

### 2.4 Effects of XIST on miRNA levels and functions

Based on the sequence analyses, XIST has been predicted to act as a competitive endogenous RNA (ceRNA) or miRNA sponge with a potential to sequester cellular miRNAs and relieve repression on their downstream mRNA targets (Salmena et al., 2011; Chen et al., 2017; Zhang et al., 2019). This ceRNA behavior can be particularly deleterious given the pleiotropic roles of miRNAs and the specificity of their targets across tissues. As such, XIST acts as a molecular sink, perturbing miRNA networks and amplifying pro-inflammatory, pro-survival, or pro-fibrotic signals depending on the context of disease.

The concept of XIST acting as a ceRNA concept was embraced by many investigators but was rightfully scrutinized due to the limitations of the sequence-based predictions of XIST interactions with miRNA and considerations of XIST and miRNA subcellular localization (Marshall et al., 2019). Although sequence based computational analyses suggested that XIST has potential to interact with 864 miRNA species, only 13 miRNA-mRNA pairs found empirical evidence for regulation by XIST as ceRNA in the lung adenocarcinoma cell lines (Marshall et al., 2019). It is likely that the tissue- and cell type-specific patterns of XIST and miRNA expression and localization, XIST occupancy by the RNA-binding proteins, cell stress and activation status alter the identity, spectrum and magnitude of the miRNA that are impacted by XIST. More comprehensive analyses are warranted to describe the cell-type specific effects of XIST on miRNA-mRNA dynamics, XIST localization, and the mode of XIST action on the miRNA.

When analyzing XIST-miRNA interactions, it is important to differentiate the direct interaction between XIST and mature miRNA versus XIST-mediated epigenetic silencing of the genes encoding pri-miRNA. The human X chromosome encodes at least 118 miRNAs (∼10% of the miRNAs identified in the human genome). Among the 62 experimentally validated X-linked miRNA, 41 are intragenic, i.e., located on the same strand as the host protein coding gene and 21 are intergenic, i.e., representing non-coding genes (Di Palo et al., 2020). These miRNA genes can be subject to XCI or may escape it. XIST-mediated effects on the X-linked miRNA genes are likely to happen in a sequence independent manner but result in a downregulatory net effect.

Canonically, XIST is characterized as a nuclear lncRNA essential for X-chromosome inactivation and XIST localization was reproducibly described as predominantly nuclear, associated with the inactivated X chromosome (Brown et al., 1992; Jonkers et al., 2008). XIST exon 7 and interaction with hnRNP U, along with the poor recognition of XIST by the RNA export factor TAP/NXF1 mediate XIST nuclear retention (Cohen and Panning, 2007; Yamada et al., 2015; Owens et al., 2025). Yet, growing evidence suggests that XIST can accumulate in the cytoplasm, where it exerts extranuclear effects under cellular stress or pathological conditions, or can even be packaged within extracellular vesicles. Fluorescent *in situ* hybridization and subcellular fractionation followed by qRT-PCR analyses demonstrated XIST translocation in response to cell activation and stress in multiple experimental systems (Shenoda et al., 2021; Cheng et al., 2024). The extranuclear interactions represent a noncanonical, post-transcriptional regulatory axis in which XIST no longer acts as an epigenetic scaffold but instead functions as a pathogenic modulator of gene expression via miRNA sequestration.

Aberrant overexpression and/or cytoplasmic translocation of XIST allow it to engage miRNAs and disrupt cellular homeostasis and contribute to pathogenesis of cancer, autoimmune diseases, and cardiovascular diseases. A specific example is XIST-mediated sequestration of miR-29c in glioma, leading to increased expression of oncogenic targets such as transcription factor specificity protein 1 (SP1) and O6-methylguanine-DNA methytransferase (MGMT) and promoting tumor progression and chemoresistance (Du et al., 2017). Thus, XIST has been regarded as an oncogene interacting with a number of tumor-suppressing miRNAs, such as miR-152, miR-101, miR-124, miR-29c, miR-29a, miR-140, miR-367/141, and miR-133a (Liu et al., 2018). Similarly, XIST-mediated sequestration of miR-34a disrupts regulatory pathways and promotes chronic inflammation in rheumatoid arthritis (Wei et al., 2023).

### 2.5 XIST-mediated miRNA sequestration in the context of CVD and related diseases

The roles of XIST-mediated miRNA sequestration in cardiovascular disease appears to be dependent on the pathological context and experimental model and require more rigorous examination (Table 2). Increased levels of XIST RNA were reported in a model of myocardial infarction, where sequestration of miR-101a-3p by XIST de-repressed transcription factor FOS and increased cardiomyocyte apoptosis (Lin et al., 2020). Two studies demonstrated increased expression of XIST in a model of cardiac hypertrophy induced by transverse aortic constriction (TAC) surgery, but suggested opposite outcomes: sequestration of miR-101 lead to increased TLR2 expression and enhanced phenylephrine-induced cardiomyocyte hypertrophy (Xiao et al., 2019), whereas sequestration of miR-330-3p increased expression of S100B and mitigated phenylephrine-induced cardiomyocyte hypertrophy (Chen et al., 2018). Unbiased analyses of the lncRNA-miRNA-mRNA regulatory networks identified blood XIST RNA levels as a biomarker for acute myocardial infarction (Zheng et al., 2022). However, the questions about specificity of this biomarker and the origin of XIST in blood after myocardial infarction have not been addressed.

**TABLE 2:** Reported examples of miRNA affected by XIST ceRNA activity in the context of CVD models.

miRNA affected by XIST	miRNA target	Disease or experimental model	Direct evidence for XIST as the miRNA sponge	References
miR-101	FOS	Mouse model of myocardial infarction, neonatal cardiomyocytes	Luc-XIST, Biotin pulldown, AGO2 pulldown, siXIST	Lin et al. (2020)
miR-101	TLR2	Mouse model of cardiac hypertrophy (TAC) and phenylephrine-induced cardiomyocyte hypertrophy	Luc-XIST	Xiao et al. (2019)
miR-330-3p	S100B	Mouse model of cardiac hypertrophy (TAC) and phenylephrine-induced cardiomyocyte hypertrophy	Luc-XIST, XIST OE, miR OE	Chen et al. (2018)
miR-214-3p	ARL2	Mouse atrial fibrillation and mouse atrial cardiomyocyte cell line HL-1	Luc-XIST AGO2 pulldown XIST OE	Yan et al. (2021)
miR-150-5p	BAX	Rat myoblast cell line H9c2	Luc-XIST AGO2 pulldown, siXIST	Zhou et al. (2020)
miR-130a-3p	PDE4D	Human cardiomyocyte cell line A16	Luc-XIST	Zhou et al. (2019)
miR-133a	SOCS2	Rat myoblast cell line H9c2	Luc-XIST XIST OE, siXIST	Li et al. (2019)

Luc-XIST, a luciferase reporter assay using the predicted miRNA, binding site within XIST, lncRNA, downstream of luciferase coding sequence, showed the inhibitory effect of the miRNA, or miR mimic.

Biotin pulldown and AGO2 pulldown, XIST RNA was detectable after using biotinylated miRNA, mimic and streptavidin or AGO2-specific antibodies.

siXIST, siRNA-mediated knockdown of endogenous XIST, downregulated the miRNA, levels.

XIST OE, XIST, overexpression downregulated the endogenous miRNA.

miR OE, miRNA, or miR mimic downregulated endogenous XIST, expression.

Decreased levels of endogenous XIST were reported in mouse atrial tissue in a model of atrial fibrillation (Yan et al., 2021). XIST levels could be partially restored by administration of extracellular vesicles produced by either intact or XIST overexpressing adipose tissue-derived mesenchymal stem cells. In this settings, XIST was shown to protect from myocardial pyroptosis by acting as ceRNA for miR-214-3p and increasing expression of a GTP-binding ARL2 (Yan et al., 2021).

Other reports showed pro-apoptotic effects of XIST in response to hypoxia in cardiomyoblast cell lines along the miR-130a-3p/PDE4D (Zhou et al., 2019), miR-133a/SOCS2 (Li et al., 2019), and miR-150-5p/Bax (Zhou et al., 2020) interactions. However, the limitations of the cell models used raise questions about the translational impact of those studies. Despite the limitations, multiple lines of evidence, such as luciferase reporter assays, RNA pulldowns, and effects of XIST overexpression on reducing the target miRNA levels, demonstrated direct interactions of XIST with the miRNA and XIST role as ceRNA. However, to establish the role of XIST as a potent ceRNA or miRNA sponge beyond any doubt, more comprehensive unbiased studies are necessary that can establish endogenous XIST and miRNA colocalization, direct interaction, with the loss-of-function and gain-of-function genetic evidence for XIST and the miRNA in question in primary cells and *in vivo*.

### 2.6 Potential extranuclear roles of XIST

The presence of XIST outside of the nucleus can be sensed by the single-stranded RNA sensors, such as TLR7 and TRL8 (which are also known to escape XCI) and trigger interferon responses and inflammation. Furthermore, extracellular XIST may act as a scaffold for RNA-binding proteins and drive female-biased autoimmunity. A recent elegant study showed that expression of an inducible Xist transgene in male mice on SJL/J genetic background leads to abnormal activation of T cells and B cells and promotes multi-organ autoimmune pathology (Dou et al., 2024). Autoimmune mice and human patients display autoantibodies that react with the XIST-protein complexes.

Another potentially important aspect of XIST translocation to the cytoplasm drew investigators’ attention with the advent of polysome profiling and translating ribosome affinity purification (TRAP) followed by RNA sequencing. Although XIST reads are often detected among the polysome or TRAP-enriched samples indicating ribosome occupancy, lack of evolutionary conserved significant open reading frame and the pattern of ribosome release indicate that XIST is a *bona fide* non-coding RNA (Guttman et al., 2013; Hou et al., 2020). Although association of XIST with ribosomes could represent evolutionary noise (Guttman et al., 2013), similar to pervasive transcription, it is possible that XIST interactions with ribosomes may regulate its extranuclear stability and availability to act as a miRNA sponge. Proximity to translation machinery may also increase the probability of direct or indirect XIST interaction with protein coding mRNA and selective regulation of translation efficiency.

Understanding the extranuclear functions of XIST not only expands our appreciation of lncRNA versatility but also presents a rationale for targeted and sex-specific therapeutic strategies using ASO, siRNAs, miRNA mimics and antagonists and TSBs. In addition to considering the direct RNA therapeutic targets, it may be necessary to consider the risks vs. benefits of engaging XIST, based on the sequence homology and possibility that XIST could be translocated into cytoplasm in the targeted cells.

## 3 Brief overview of RNA therapeutics for cardiovascular diseases

RNA therapeutics can be broadly defined as a class of drug modality to prevent, change the course, or cure specific diseases by using or targeting specific RNA molecules associated with the disease pathogenesis. RNA therapeutics include oligonucleotide therapeutics, such as antisense oligonucleotides (ASO), small interfering RNA (siRNA), miRNA mimics or antagonists, splicing regulators, RNA aptamers, and large RNA molecules, such as mRNA, self-amplifying RNA, or circular RNA that can be used as vaccines or for protein replacement approaches (Wang et al., 2025). Key advantages of RNA therapeutics include rational design and targeted action using RNA sequence specificity and/or structure, ability to reach intracellular protein targets through regulation of their gene expression at transcriptional and post-transcriptional levels, regulated immunogenicity or lack of it due to incorporation of modified nucleotides, rapid development and scalability relying on greatly enhanced manufacturing processes and capacity, and a potential for combination therapies. Although RNA therapeutics still face challenges in delivery to specific cells and tissues, relatively short half-life, potential for inflammatory responses, manufacturing complexity and high cost of production, as well as possible off-target effects, they hold promises for prevention and treatment of a variety of conditions, including cardiovascular diseases.

### 3.1 Oligonucleotides: from anti-sense to miRNA mimics and antagonists

**ASO** are short 20–24 nucleotide long, synthetic strands of nucleic acids designed to hybridize to specific sequences on target mRNAs through Watson–Crick base pairing, whereby each base on the ASO pairs with its complementary nucleotide on the RNA target to form a stable duplex (Crooke et al., 2021a). ASO are typically designed to downregulate gene expression by inducing degradation of target RNAs, including mRNAs and lncRNAs.

The first approved ASO was fomivirsen (developed by Isis Pharmaceuticals and marketed as Vitravene), which was used to target cytomegalovirus mRNA for treatment of AIDS-related cytomegalovirus-induced retinitis (Roehr, 1998). Although the efficacious use of highly active antiretroviral therapy led to eventual withdrawal of fomivirsen from the markets, its initial success demonstrated ASO potential for clinical applications. Most of the other ASO that are approved or in development target rare genetic diseases, such as Duchenne muscular dystrophy, spinal muscular atrophy, hereditary transthyretin amyloidosis, thus and familial chylomicronemia syndrome (Crooke et al., 2021a). The first ASO used for treatment of cardiovascular disease was mipomersen sodium (developed by Isis Pharmaceuticals and marketed as Kynamro™ by Genzyme Corporation), which received FDA approval in 2013 as an adjunct to lipid-lowering medications and diet in patients with homozygous familial hypercholesterolemia. Mipomersen targets apolipoprotein B-100 translation to reduce LDL cholesterol and other atherogenic lipoproteins. However, the risks of adverse effects, mainly liver toxicity, led to rejection by EMA and subsequent withdrawal of FDA approval in 2019. Volanesorsen (developed by Ionis Pharmaceuticals and its subsidiary Akcea Therapeutics and marketed as Waylivra) is another lipid lowering ASO, acting by inhibition of apolipoprotein C-III translation. It received EMA approval in 2019 as an adjunct to diet in adult patients with genetically confirmed familial chylomicronemia syndrome and at high risk for pancreatitis but was rejected by FDA on concerns due to platelet losses and risk of bleeding (Author Anonymous, 2018). Improved ASO design for effective mRNA targeting in combination with modifications to ASO backbone and sugars and specific delivery lend optimism to the developers of ASO therapeutics maintaining a large pipeline of clinical candidates for cardiovascular and metabolic diseases as well as other indications (Crooke et al., 2021a).

These clinical milestones highlight how far ASO therapeutics have progressed, but their success ultimately depends on chemical innovations that improve stability, delivery, and potency. Chemical modifications to ASO targeting XIST may help us better understand its biological functions and explore the therapeutic potential for neutralizing XIST activity. Central among these is the phosphorothioate (PS) backbone modification, which enhances nuclease resistance and enables efficient RNase H1 recruitment. Phosphorothioate (PS) modifications enhance ASO resistance to nuclease degradation and facilitate recruitment of endogenous RNA-degrading enzymes such as RNase H1. In PS-modified oligos, one of the non-bridging oxygen atoms in the phosphate backbone is replaced by a sulfur atom, improving resistance to nuclease degradation while maintaining the overall structure of natural nucleic acids.

Among the most effective ASO designs for mRNA transcripts are gapmer ASOs, which feature a central DNA “gap” flanked by chemically modified ribonucleotides, such as 2′-O-methyl or 2′-O-methoxyethyl (MOE). This modification also enhances protein binding and facilitates cellular uptake (Crooke, 2017). In the gapmer configuration, PS oligonucleotides are designed with a central DNA region flanked by chemically modified nucleotides, enabling them to hybridize to complementary RNA sequences. Upon binding, the resulting RNA–DNA duplex is recognized by RNase H1, an endogenous enzyme that selectively cleaves the RNA strand, leading to efficient and catalytic transcript degradation (Crooke et al., 2021b). This mechanism enables potent and durable knockdown of both nuclear and cytoplasmic RNA targets. PS-modified ASO, such as inotersen, have demonstrated clinical efficacy and regulatory approval, establishing phosphorothioate-based designs as a validated platform for RNA-targeted therapeutics (Gales, 2019).

The overall effectiveness of gapmer ASOs depends on several factors, including the accessibility of the target site, the thermodynamic stability of the ASO–RNA duplex, and the cellular abundance of RNase H1 (Crooke, 2017). This mechanism has been successfully applied to deplete nuclear lncRNAs such as MALAT1 and NEAT1, underscoring the suitability of gapmers for targeting overexpressed nuclear transcripts like XIST (Amodio et al., 2018). The tissue-selective delivery of ASOs via conjugates or lipid nanoparticles is an active area of research that could further enhance safety and specificity.

Hybridization-dependent and independent toxicities remain major challenges in oligonucleotide therapeutics. Hybridization-dependent effects arise from unintended base-pairing with partially complementary transcripts, leading to off-target mRNA degradation or translational inhibition. In contrast, hybridization-independent toxicities reflect non-sequence-specific interactions of chemically modified oligonucleotides with intracellular or extracellular proteins, membranes, or nucleic acid–sensing receptors, which can result in cytotoxicity, immune activation, or complement interference. Recent advances have mitigated these risks through rational chemical optimization and delivery engineering. Sugar modifications such as 2′-O-methyl, 2′-O-methoxyethyl (MOE), and locked nucleic acid (LNA) substitutions increase nuclease resistance and binding affinity while dampening innate immune responses (Crooke et al., 2021a). Selective phosphorothioate (PS) patterning and alternative backbones, such as phosphorodiamidate morpholino (PMO), peptide nucleic acid (PNA), and triazole-linked scaffolds, further reduce protein-binding–mediated toxicities (Shen et al., 2019). Concurrently, targeted delivery strategies, including N-acetylgalactosamine (GalNAc) conjugates for hepatocyte uptake, lipid nanoparticles (LNPs) for systemic or localized administration, and peptide or antibody conjugates for extrahepatic targeting, limit non-specific biodistribution and lower exposure of sensitive tissues (Tang and Khvorova, 2024). Collectively, these advances demonstrate that chemical and formulation innovations can substantially mitigate both hybridization-dependent and -independent toxicities, providing a rational path toward safer and more selective RNA-targeted therapeutics, including future strategies directed against cytoplasmic XIST.

**RNA editing oligonucleotides and guide RNA (gRNA)** represent a novel and promising RNA therapeutics approach relying on adenosine deaminases acting on RNA (ADARs) to correct disease-causing G-A missense and non-sense mutations at the mRNA level. The oligonucleotides and gRNA are usually designed as antisense RNA flanking the mutation in the target mRNA with the cytosine opposed to the adenine. The mismatch in a preferred context recruits and activates ADAR complex that replaces adenine with inosine (A-to-I editing) resulting in a functional correction of the mutation since inosine can be interpreted as guanosine during translation (Booth et al., 2023). An investigational RNA editing oligonucleotide WVE-006 is in clinical development by Wave Life Sciences for treating alpha-1 antitrypsin deficiency. Several other candidates are expected to enter clinical trials in 2025, including AX-1412 by ProQR Therapeutics who aims to use RNA editing for expression of protective allele of B4GALT1 enzyme, a galactosyltransferase that attaches galactose to glycoprotein substrates including lipoproteins and fibrinogen and that is associated with reduced risk of CVD (Montasser et al., 2021; Mullard, 2024). In addition, other groups are developing alternative approaches to RNA editing that do not rely on the endogenous RNA editing enzymes of ADAR family, but employ the RNA editing activity of CRISPR/Cas13 orthologues (Birgaoanu et al., 2023).

**Small interfering RNA (siRNA)** are typically 21–23 nucleotide double-stranded RNA molecules that are processed by Dicer and incorporated into the RNA-induced silencing complex (RISC). Within RISC, the guide strand directs the Argonaute (AGO) protein (most commonly AGO2) to complementary mRNA targets, leading to endonucleolytic cleavage and subsequent degradation of the transcript (Setten et al., 2019; Iwakawa and Tomari, 2022; Tang and Khvorova, 2024). In 2018, patisiran (Onpattro) developed by Alnylam Pharmaceuticals became the first FDA approved siRNA drug to treat hereditary transthyretin-mediated amyloidosis-validating RNAi approach. Since then, additional siRNA-based medications such as givosiran and lumasiran have received approval, with vutrisiran following in 2022, underscoring expanding momentum in rare genetic diseases (Scott, 2020; Padda et al., 2024).

**miRNA mimics and miRNA antagonists in therapeutic development**. MicroRNAs (miRNAs) are critical post-transcriptional regulators by binding to complementary sequences in the 3′ untranslated regions (3′UTRs) of target mRNAs, leading to translational repression or transcript degradation. A single miRNA can regulate multiple mRNA transcripts enabling coordinated post-transcriptional repression of functionally related genes and fine-tuning entire gene networks. Dysregulation of miRNA expression has been implicated in a wide array of diseases, including cancer, cardiovascular disorders, autoimmune diseases, and neurodegeneration (Ha, 2011; Juźwik et al., 2019). This has prompted the development of miRNA-based therapeutics, primarily falling into two categories: miRNA mimics and miRNA antagonists (anti-miRs) (Seyhan, 2024).

miRNA mimics are synthetic double-stranded RNAs designed to restore the function of downregulated miRNAs that normally act as tumor suppressors or regulators of cellular homeostasis. By replenishing these lost regulatory activities, mimics can suppress oncogenic or pro-inflammatory gene networks. For example, miR-34 and let-7 mimics have been evaluated in preclinical and early clinical studies targeting cancers and fibrotic diseases (Elliot et al., 2019; Hong et al., 2020).

miRNA antagonists, also known as antagomirs or anti-miRs, are chemically stabilized single-stranded oligonucleotides that bind and sequester overactive or pathogenic miRNAs, thereby preventing them from repressing their mRNA targets. A prominent example is miravirsen, an LNA-modified anti-miR against miR-122, which demonstrated antiviral efficacy in hepatitis C virus (HCV) infection by blocking a host miRNA essential for viral replication (Liu et al., 2016). While these strategies underscore the therapeutic potential of directly modulating miRNAs, clinical translation has been limited by off-target effects, delivery barriers, and limited specificity (Seyhan, 2024).

**Target site blockers (TSBs)** are an emerging and modular class of oligonucleotide therapeutics designed to sterically inhibit specific miRNA–mRNA interactions without affecting the overall miRNAs levels (Gilot and Galibert, 2018; De Santi et al., 2020; Fitzsimons et al., 2023). Unlike ASOs or siRNAs, TSBs modulate gene expression by selectively blocking miRNA binding sites on target RNAs. The functional outcome of a TSB depends on its chemical backbone. TSBs in the phosphorothioate (PS-TSB) gapmer configuration promote RNase H1–mediated degradation of the target RNA, thereby reducing transcript levels. In contrast, morpholino-based TSBs are non-degradative; they sterically block miRNA binding while preserving transcript integrity, due to their uncharged, nuclease-resistant backbone composed of morpholine rings and phosphorodiamidate linkages (PMO). Thus, ASOs and TSBs offer complementary therapeutic strategies: ASOs degrade target RNAs broadly, while TSBs, particularly those with morpholino backbones, preserve the transcript and disrupt only specific pathogenic interactions.

### 3.2 Synthetic mRNA, self-amplifying RNA, and circular RNA

The success of RNA-based vaccines and the growing understanding of RNA biology have catalyzed the development of diverse RNA modalities beyond traditional mRNA. Among these, synthetic mRNA, self-amplifying RNA (saRNA), and circular RNA (circRNA) represent innovative platforms with unique pharmacological properties and therapeutic potential (Jones et al., 2024).

**Synthetic mRNA** therapeutics typically consist of in vitro–transcribed (IVT) messenger RNA encoding a therapeutic protein of interest. These constructs incorporate modified nucleotides (e.g., N1-methylpseudouridine), optimized UTRs, and a 5′ cap and 3′ poly(A) tail to enhance stability, translational efficiency, and immune evasion (von der Haar et al., 2023). The success of mRNA vaccines for COVID-19 (e.g., BNT162b2, mRNA-1273) demonstrated the rapid scalability, potency, and safety of this platform (Parhiz et al., 2024). Many synthetic mRNA candidate therapeutics are in development for the following applications.

#### 3.2.1 Cancer vaccines

Harnessing its flexibility and scalability, synthetic mRNA is increasingly used in cancer immunotherapy, with a strong focus on personalized vaccine development. These vaccines are engineered to encode tumor-associated antigens (TAAs) or patient-specific neoantigens, allowing host antigen-presenting cells to translate the mRNA and present immunogenic epitopes that stimulate robust CD4^+^ and CD8^+^ T cell responses against tumor cells (Sun et al., 2023; Sethna et al., 2025). This approach has demonstrated promising preclinical efficacy and is being evaluated in multiple clinical trials targeting melanoma, lung, prostate, and other solid tumors (Beck et al., 2021).

#### 3.2.2 Protein replacement

Synthetic mRNA offers a promising approach for protein replacement in genetic and metabolic diseases characterized by loss-of-function mutations. IVT mRNAs have been developed to encode functional proteins such as cystic fibrosis transmembrane conductance regulator (CFTR) for cystic fibrosis (Rowe et al., 2023) or coagulation factor VIII hemophilia A (Chen et al., 2020). These mRNA constructs are engineered with optimized untranslated regions (UTRs), codon usage, and modified nucleotides to enhance stability and translation while minimizing immunogenicity. Preclinical and early clinical studies demonstrate that synthetic mRNA can restore physiological protein levels in target tissues including heart, supporting its therapeutic potential as a non-integrating, versatile modality for protein replacement (Qin et al., 2022).

#### 3.2.3 Promoting tissue repair and regeneration

The application of synthetic mRNA has shown promise in regenerative medicine, offering several advantages over DNA-based strategies, such as avoiding genomic integration and enabling rapid cytoplasmic expression (Hınçer et al., 2023). Its programmability, rapid expression kinetics, and safety profile, due to the absence of genomic integration, make it especially attractive for tissue repair strategies across organ systems (Steinle et al., 2021) including CVD (Zangi et al., 2013; Magadum et al., 2019; Żak and Zangi, 2025). Engineered mRNAs have been designed to promote regeneration in cardiac, hepatic, and musculoskeletal tissues. For example, VEGF-A mRNA, delivered via LNPs, has been shown to enhance angiogenesis and cardiac function in preclinical models of myocardial infarction (Carlsson et al., 2018). In the liver, synthetic mRNA encoding hepatocyte growth factor (HGF) or other regenerative cues has demonstrated the capacity to restore hepatic function after injury (Cacicedo et al., 2022). Naked mRNA encoding VEGF-A (AZD8601, co-developed by Moderna and AstraZeneca) was one of the most advanced mRNA therapeutic candidates as it showed promise in a Phase 2a study in patients undergoing coronary artery bypass grafting (Anttila et al., 2023). Advanced delivery strategies are central to these applications, including lipid-based nanoparticles, hydrogels, electroporation, and scaffold-assisted delivery, which facilitate cell-type-specific uptake and sustained expression at the target site.

**Self-Amplifying RNA (saRNA)** (or self-replicating RNA) represents an advanced RNA therapeutic platform engineered to amplify protein expression within the cytoplasm by harnessing elements from alphavirus genomes. Unlike conventional mRNA, saRNA retains the viral replicase (RNA-dependent RNA polymerase), enabling intracellular self-replication of both the original transcript and a subgenomic mRNA encoding the therapeutic payload, while the structural protein genes are replaced by the gene of interest (Vallet and Vignuzzi, 2025).

This self-replicative mechanism results in amplified and sustained protein production from significantly lower doses of RNA. Notably, preclinical studies of saRNA-based COVID-19 vaccines in animals demonstrated robust antigen-specific immune responses comparable or superior to conventional mRNA at reduced doses (McKay et al., 2020; Silva-Pilipich et al., 2024; Yang et al., 2025). The saRNA vaccine for COVID-19 ARCT-154 (KOSTAIVE®) developed by Arcturus Therapeutics in collaboration with CSL received approval in Japan and Europe after demonstrating superior immunogenicity and antibody persistence for 12 months compared to the conventional mRNA COVID-19 vaccine BNT162b2 (Oda et al., 2024).

The dose-sparing and durable expression characteristics of saRNA make it especially attractive for vaccine and therapeutic applications in chronic infectious diseases or cancer, which may require extended expression of single or multiple antigens with immune-modulating payload to induce robust antibody and T cell-mediated immune responses (Yarovinsky et al., 2019; Lundstrom, 2021; Alvero et al., 2024; Chen et al., 2024; Vallet and Vignuzzi, 2025). Thus, saRNA represents a promising frontier for cost-effective, scalable RNA therapeutics that leverage catalytic amplification to maximize efficacy.

**Circular RNA (circRNA)** represents a novel and increasingly explored class of RNA therapeutics characterized by their covalently closed-loop structure, which lacks 5′ and 3′ ends. This topological feature makes them intrinsically resistant to exonuclease-mediated degradation, significantly extending their intracellular half-life compared to linear mRNA counterparts (Chen, 2016; Liu et al., 2019).

Engineered circRNAs can be synthetically designed to include internal ribosome entry sites (IRESs) or N6-methyladenosine (m6A) motifs that facilitate cap-independent translation, thereby enabling sustained and tunable protein production without the need for a canonical 5′ cap structure (Wesselhoeft et al., 2018). These properties make circRNAs particularly attractive for protein replacement therapies, where prolonged protein expression is desired, and for next-generation vaccines, in which circRNA constructs can serve as durable antigen sources that persist long enough to elicit robust immune responses.

Recent preclinical studies have demonstrated that circRNA-based platforms can achieve robust and prolonged protein expression *in vivo* and elicit potent adaptive immune responses, validating their potential as therapeutic agents (Qu et al., 2022). Their circular configuration also improves thermal and enzymatic stability, supporting ease of formulation, storage, and distribution—key advantages for global therapeutic deployment. As synthetic biology and RNA engineering techniques advance, circRNA therapeutics are poised to complement and potentially surpass linear mRNA approaches in applications requiring extended expression windows, reduced dosing frequency, and improved biostability.

Together, these next-generation RNA platforms extend the therapeutic potential of RNA beyond transient expression, offering novel solutions for persistent expression, enhanced stability, and immunological control. Their modularity and adaptability further support development across diverse disease areas, including infectious diseases, cancer, cardiovascular conditions, and rare genetic disorders.

Organ accessibility, biodistribution, and off-target exposure remain central barriers to the clinical translation of siRNA and miRNA therapeutics. Despite major advances in RNA chemistry and design, the efficient and selective delivery of small RNA drugs to extrahepatic tissues remains an unresolved challenge. siRNA and miRNA mimics or inhibitors must overcome rapid renal clearance, serum nuclease degradation, and endosomal sequestration to achieve therapeutic concentrations in the intended cell type. The success of hepatocyte-targeted GalNAc–siRNA conjugates demonstrates the potential of ligand-directed delivery, but similar strategies for lung, heart, and immune tissues are still under active development. Lipid nanoparticle (LNP) formulations have expanded the therapeutic reach of RNA modalities by enabling encapsulation, protection, and controlled release of double-stranded or single-stranded oligonucleotides (Mashima and Takada, 2022). Recent innovations, including ionizable lipid optimization, biodegradable ester linkages, and surface PEGylation, have improved tissue tropism, reduced systemic immunogenicity, and enhanced endosomal escape efficiency (Nair et al., 2014). Complementary progress in localized delivery methods, such as intratracheal, intravitreal, and topical administration, has shown that restricting exposure to the disease site can markedly increase specificity and reduce systemic side effects (Roberts et al., 2020). Nevertheless, biodistribution heterogeneity, innate immune activation, and complement-mediated clearance remain limiting factors. Continued refinement of LNP chemistry, combined with emerging polymeric and peptide-based carriers, is expected to enhance the precision and safety of RNA drug delivery. Within this broader context, any therapeutic targeting cytoplasmic XIST will require similar delivery innovations—prioritizing tissue specificity, endosomal release, and controlled biodistribution—to achieve selective modulation of its pathogenic functions without systemic toxicity.

## 4 XIST as a target of RNA therapeutics

XIST is an important but challenging target for drug development for treatment of inherited X-linked disorders in females. Downregulation of XIST-mediated XCI by using XIST-specific ASO, repressive miRNA or inhibitors of XIST activity may reawaken the healthy alleles on the inactivated X chromosome (Carrette et al., 2018; Aguilar et al., 2022; Lou et al., 2025). This strategy is being actively pursued with the goal of finding treatment for Rett syndrome, caused by loss-of-function mutations of *MECP2* gene. The initial proof of concept studies included a combination of XIST ASO gapmers and a short-term 5-aza-2′-deoxycytidine (Aza) treatment to induce DNA demethylation and open the chromatin structures on the inactive X chromosome (Carrette et al., 2018). This approach resulted in up to 30,000-fold increase of MECP2-luciferase reporter expression from the inactive X chromosome in the clonal hybrid cell line and reactivation of multiple genes in mouse female fibroblasts (Carrette et al., 2018). The same group pursued a screen of 50,000-compound library to identify agents that bind to the repeat region A of XIST and found that at least one of them displaces Polycomb repressive complex 2 (PRC2) and SPEN RNA-binding proteins that facilitate XIST-mediated repressive histone modifications and XCI (Aguilar et al., 2022). The compound X1 weakens XIST interaction with PRC2 and SPEN in cells at 10 µM concentration and disrupts the XCI in embryonic cells and mice (Aguilar et al., 2022). The authors anticipate that medicinal chemistry of compound X1 may improve its potency and specificity, thus raising hope for development of therapy for Rett syndrome and other X-linked diseases. Another group recently discovered that miR-106a binds to XIST repeat A region and that this interaction is essential for association of XIST with the inactive X chromosome. Depletion of miR-106a with a specific microRNA sponge decreased binding of several RNA-biding proteins, including HNRNPK, to XIST (Lou et al., 2025). Delivery of the miR-106a-specific miRNA sponge using adeno-associated virus increased MCEP2 expression in the brain of mice and improved the phenotype in the mouse model of Rett syndrome.

The rationale for targeting XIST in cancers is based on hypothesis and empirical data suggesting that modulating XIST expression and/or its miRNA-regulatory functions may inhibit the progression of bladder, colorectal and lung cancers. Atractylenolide II and Platycodin D, which are apoptosis inducers used in Chinese and East Asian traditional medicine to treat cancers, showed inhibitory activity on XIST expression and reversed the inhibitory effects of XIST on miR-30a-5p and miR-335, respectively (Li et al., 2022). However, the exact mechanism of action for these compounds with relation to XIST downregulation has not been elucidated.

The pathological contexts when XIST is mis-localized to the cytoplasm (e.g., certain cancers or myocardial infarction) prompt consideration of targeting XIST with gapmer ASOs that are particularly effective due to their compatibility with RNase H1 activity in the nucleus and cytoplasm. Nevertheless, global knockdown of XIST using ASOs requires careful consideration of tissue specificity and dosage, especially in female patients where complete loss of XIST function could compromise X-chromosome dosage compensation. As such, ASO strategies may be most appropriate for conditions where XIST is ectopically expressed in normally silent tissues (e.g., tumors, injured myocardium), or where its canonical XCI role is dispensable (Lin et al., 2020; Zong et al., 2020). For instance, in glioma models, ASO-mediated knockdown of XIST could theoretically restore miR-29c activity, thereby decreasing SP1 and MGMT expression, two key mediators of chemoresistance (Yuan et al., 2022).

In the context of diseases where XIST mis-localization or aberrant miRNA sponging contributes to pathology, TSBs offer a targeted means of intervention by blocking miRNA binding sites on either mRNAs or lncRNAs like XIST. This is particularly advantageous for transcripts like XIST, whose physiological roles in X-chromosome inactivation and nuclear architecture make global silencing undesirable. Instead, PMO-TSBs enable selective disruption of pathological interactions while maintaining normal functions (Yokota et al., 2009). For example, a PMO-TSB targeting the miR-101a-3p binding site on XIST could restore miR-101a-3p availability to suppress FOS, thereby reducing cardiomyocyte apoptosis following myocardial infarction (Lin et al., 2020). This occupancy-based mechanism allows for precision modulation of disease-relevant RNA networks without neutralizing miRNAs globally—an important distinction given their widespread regulatory roles. With increasing mechanistic insight into the XIST–miRNA–mRNA axis across diverse diseases, and growing advances in oligonucleotide chemistry, these tools hold significant translational promise for XIST-related disorders.

As safety consideration are essential at all stages of drug development, it is imperative to consider the risks associated with the off-target effects, whether the drugs in development are intended to modulate XCI and XIST or not. Evidently, targeting XIST for degradation or blocking its epigenetic activity is likely to result in X-chromosome-wide escape from XCI, albeit the effect is likely to be dose- and time-course dependent. Degradation of XIST is also likely to result in diminishing its miRNA sponge activity outside of the nucleus, resulting in pleiotropic effects on gene expression at post-transcriptional levels (mRNA stability and/or translation). On the other hand, interventions resulting in expulsion of XIST from the Xi may result in translocation of XIST to cytoplasm, resulting in downregulation of multiple miRNA and possible activation of inflammatory signals triggered by TLR7/TLR8 signaling and interferon responses, which may proceed to autoimmune reactions. Inclusion of female cells and animals at the preclinical stages and female patients in the early clinical trial stages, with appropriate readouts for XCI, XIST expression and activity, and X-linked gene expression is likely to inform the investigators of the risks and potential adverse effects due to off-target effects.

## 5 Conclusions and future directions

The knowledge accumulated to date indicates that XIST is a major regulator of gene expression at the epigenetic level for X-linked genes via XCI. The roles of XIST in post-transcriptional regulation of gene expression via sequestration of specific miRNAs require further comprehensive investigation and solid evidence for XIST and miRNA colocalization and direct interactions. It is likely that the newly developed RNA therapeutics may impact XIST functions, either by rational design or due to off-target effects. We propose considering the following steps and criteria when developing or evaluating responses to RNA therapeutics for CVD in women.

- Use computational sequence and structure analyses tools, XIST affinity/binding assays, and assessment of XIST expression levels and subcellular localization to determine whether the RNA therapeutics are likely to interact with XIST directly or via its partners, such as the RNA binding proteins or miRNA.
- When selecting and prioritizing the targets for RNA therapeutics, assess whether the associated genes regulating target expression and activity are X-linked and whether they are subject to XCI or escape it.
- Since women are mosaics with respect to the gene expression from X-chromosome, consider using single cell or spatial analyses or high-throughput analyses of XCI and escape when assessing the responses of females to RNA therapeutics.
- When designing the clinical trials including both sexes, assess the probability of enrolling, knowingly or unknowingly, males that have 47,XXY karyotype (with or without obvious characteristics of Kleinfelter syndrome) and are likely to express XIST.

We hope that the risks of adverse effects for novel RNA therapeutics could be reduced, especially for women, through consideration of the pleiotropic effects of XIST and implementation of the proposed measures into drug development.

## Data Availability

The original contributions presented in the study are included in the article/supplementary material, further inquiries can be directed to the corresponding author.

## Footnotes

1 Marsupial mammals exert X chromosome dosage compensation via imprinted XCI, where the paternally inherited X chromosome (Xp) is suppressed, but the maternally inherited X chromosome (Xm) remains fully active (Grant et al., 2012). Long non-coding RNA Rsx mediates X chromosome inactivation in marsupials, but it is evolutionally not related to XIST (Whitworth and Pask, 2016). Monotremes (platypus and echidnas) have multiple X and Y chromosomes and exert gene dosage compensation at individual genes rather than at whole chromosome level (Whitworth and Pask, 2016).
